# Supplementary material for: Novel Driver Strength Index highlights important cancer genes in TCGA PanCanAtlas patients
Source: PeerJ. 2022 Aug 11;10:e13860. doi: 10.7717/peerj.13860 (PMC9375969; doi:10.7717/peerj.13860)
Supplement: Supplemental Information 2 [file peerj-10-13860-s002.zip › COHORTS/genes plots/cohorts/2021_8_16_14_9_distribution_cohorts_males_PCPG.pdf]

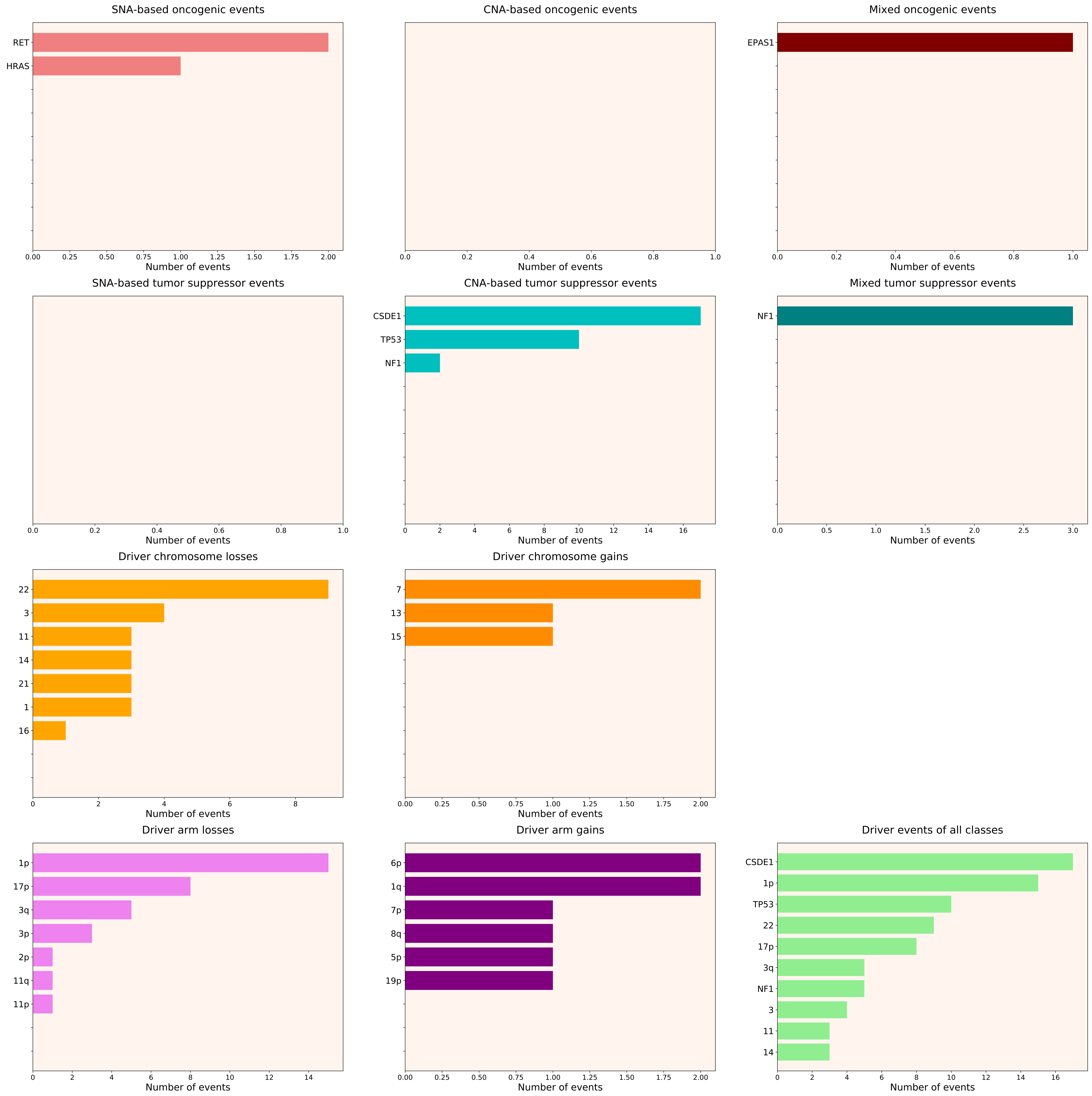
SNA-based oncogenic events
RET
HRAS
Number of events
CNA-based oncogenic events
Number of events
Mixed oncogenic events
EPAS1
Number of events
SNA-based tumor suppressor events
Number of events
CNA-based tumor suppressor events
CSDE1
TP53
NF1
Number of events
Mixed tumor suppressor events
NF1
Number of events
Driver chromosome losses
22
3
11
14
21
1
16
Number of events
Driver chromosome gains
7
13
15
Number of events
Driver arm losses
1p
17p
3q
3p
2p
11q
11p
Number of events
Driver arm gains
6p
1q
7p
8q
5p
19p
Number of events
Driver events of all classes
CSDE1
1p
TP53
22
17p
3q
NF1
3
11
14
Number of events
